# Phenotype, Body Composition, and Prediction Equations (Indian Fatty Liver Index) for Non-Alcoholic Fatty Liver Disease in Non-Diabetic Asian Indians: A Case-Control Study

**DOI:** 10.1371/journal.pone.0142260

**Published:** 2015-11-24

**Authors:** Surya Prakash Bhatt, Anoop Misra, Priyanka Nigam, Randeep Guleria, M. A. Qadar Pasha

**Affiliations:** 1 Diabetic Foundation (India) and National Diabetes Obesity and Cholesterol Foundation (N-DOC), New Delhi, India; 2 Department of Pulmonary Medicine and Sleep Disorders, All India Institute of Medical Sciences, New Delhi, India; 3 Fortis C-DOC Center of Excellence for Diabetes, Metabolic Diseases, and Endocrinology, B 16, Chirag Enclave, New Delhi, India; 4 Council of Scientific & Industrial Research-Institute of Genomics and Integrative Biology, Delhi, India; University of Kansas Medical Center, UNITED STATES

## Abstract

**Objective:**

In this study, we have attempted comparison of detailed body composition phenotype of Asian Indians with non-alcoholic fatty liver disease (NAFLD) *vs*. those without, in a case controlled manner. We also aim to analyse prediction equations for NAFLD for non-diabetic Asian Indians, and compare performance of these with published prediction equations researched from other populations.

**Methods:**

In this case-control study, 162 cases and 173 age-and sex-matched controls were recruited. Clinical, anthropometric, metabolic, and body composition profiles, and liver ultrasound were done. Fasting insulin levels, value of homeostasis model assessment of insulin resistance (HOMA-IR), and serum high sensitive C-reactive protein (hs-CRP) levels were evaluated. Multivariate logistic and linear regression analyses were used to arrive at prediction equations for fatty liver [Indian fatty liver index (IFLI)].

**Results:**

As compared to those without fatty liver, those with fatty liver exhibited the following; Excess dorsocervical fat (‘Buffalo hump’), skin tags, xanthelasma, ‘double chin’, arcus; excess total, abdominal and subcutaneous adiposity, and high blood pressure, blood glucose, measures of insulin resistance (fasting insulin and HOMA-IR values), lipids and hs-CRP levels. Two prediction equations were developed; Clinical [Indian Fatty Liver Index-Clinical; IFLI-C]: 1(double chin) +15.5 (systolic blood pressure) +13.8 (buffalo hump); and IFLI-Clinical and Biochemical (CB): serum triglycerides+12 (insulin)+1(systolic blood pressure) +18 (buffalo hump). On ROC Curve analysis, IFLI performed better than all published prediction equations, except one.

**Conclusion:**

Non-diabetic Asian Indians with NAFLD researched by us were overweight/obese, had excess abdominal and subcutaneous fat, multiple other phenotypic markers, had higher insulin resistance, glycemia, dyslipidemia and subclinical inflammation than those without. Prediction score developed by us for NAFLD; IFLI-C and IFLI-CB, should be useful for clinicians and researchers.

## Introduction

Non-alcoholic fatty liver disease (NAFLD) includes a spectrum of liver disorders characterized by accumulation of hepatic fat in absence of significant alcohol consumption (<20 gm/day) and other causes of liver diseases. It is most common cause of asymptomatic elevation of liver enzymes worldwide [1]. If fat accumulation continues, inflammation ensues in liver parenchyma, termed as non-alcoholic steatohepatitis (NASH), which has potential of progressing to cirrhosis.

Estimates based on imaging and autopsy studies suggest that about 20% to 30% of adults in the United States and other Western countries have NAFLD [2–4]. According to available sparse data in India, the prevalence of NAFLD ranges from 15–32% [5, 6]; variations in estimates are due to rural/urban habitat, socio-economic stratum, and varying dietary habits in different regions of country. Some data indicate that hepatic triglyceride content (estimated by proton magnetic resonance spectroscopy) of Asian Indians living in USA is more when compared to white Caucasians [7].

Several studies have suggested the associations of NAFLD with obesity, abdominal obesity, dysglycemia and other components of the metabolic syndrome [8]. Excess of body fat, mainly abdominal fat [9], is related to NAFLD [10]. Lin *et al* [11] reported that the waist circumference (WC) is better than body mass index (BMI) for predicting liver steatosis in Taiwanese subjects. Fallo *et al* [12] studied 86 hypertensive obese adults (48 NAFLD and 38 controls) and showed WC as predictor for NAFLD. Damaso *et al*. [10] reported that the group of adolescents with NAFLD had significantly higher values of BMI, visceral and subcutaneous fat, insulin, and homoeostasis modal assessment for insulin resistance (HOMA-IR) in both genders, compared with control subjects living in Brazil.

It is now well established that Asian Indians have higher levels of body fat, more abdominal adipose tissue, less lean body mass (LBM) and higher magnitude of insulin resistance than American and European subjects [13, 14]. Some data suggest that fat deposition at ectopic places (nape of the neck, excess dorsocervical fat; ‘buffalo hump’; excess fat below chin; ‘double chin’, liver) correlates with the metabolic syndrome in Asian Indians [15]. Overall a phenotype of excess body fat, low muscle mass, and fat deposition, and ectopic fat deposition is characteristic for Asian Indians [16].

It is important to identify those at risk for development of NAFLD, so that appropriate investigations could be applied for evaluation and diagnosis and for management. An optimal prediction formula for NAFLD should include simple clinical parameters which could be used by clinicians. In this context, several prediction equations have been researched in other populations; North American [17], Finnish [18] and Italian [19] population.

While others and our group have investigated phenotype and body composition of diabetic and non-diabetic Asian Indians [20], detailed studies have not been carried out in persons with NAFLD. In this study, we have attempted comparison of phenotype of those with fatty liver with those without, in a case controlled manner. Further, we present prediction equations for Asian Indians, and compare performance of these prediction equations with published prediction equations from other populations.

## Methodology

### Subjects

In this case control study, we recruited a 335 overweight/obese (238 males and 97 females) subjects (BMI ≥23kg/m^2^), 162 (129 males and 33 females) with NAFLD (cases) and 173 (109 males and 64 females) without NAFLD (controls) at two clinical sites [Fortis Hospital and All India Institute of Medical Sciences, New Delhi, India] between May 2009 and October, 2014. The study was approved by the Institutional ethics committee at Fortis Hospital and All India Institute of Medical Sciences, New Delhi, India and written informed consent was obtained. NAFLD was defined by liver ultrasonography in those with alcohol intake of less than 20 gram/day [14]. Subjects with known type 2 diabetes mellitus (T2DM), cardiovascular disease (CVD), presence of other liver diseases (alcoholic liver disease, hepatitis virus infection, autoimmune hepatitis, primary biliary cirrhosis obstruction, drug-induced liver damage etc), severe organ damage in other organs, human immunodeficiency virus infection, pregnancy, lactation, or any pro-inflammatory state and patients on statins or fenofibrate were excluded from the study.

### Clinical and Anthropometric measurements

Assessment of ‘buffalo hump’, ‘double chin’, acanthosis nigricans, skin tags or acrochordon and xanthelasma was done as described previously [15]. Blood pressure was measured by a standard mercury sphygmomanometer (Industrial Electronic and Allied Products, Pune, India) as previously [21]. Height, weight, WC, hip circumference (HC), mid arm circumference (MAC), mid thigh circumference (MTC), neck circumference (NC) and skinfold thickness at 6 sites (triceps, biceps, anterior axillary, suprailiac, subscapular and lateral thoracic) were measured according to standard protocols [22]. Subscapular: triceps skinfold thicknesses, and central (sum of subscapular and suprailiac): peripheral skinfold thicknesses (sum of biceps and triceps) were calculated.

### Biochemical analysis

Fasting blood samples were analyzed for fasting blood glucose (FBG), and post-prandial (2 hrs after meals) blood glucose, total cholesterol (TC), triglycerides (TG), high-density lipoprotein cholesterol (HDL-C), very low-density lipoprotein (VLDL-C), alkaline phosphatase (ALK), aspartate transaminase (AST), alanine transaminase (ALT) and gamma-glutamyl transpeptidase (GGT) as previously [9]. Fasting insulin levels were measured using radioimmunoassay (RIA) kits (Immunotech, France) [5]. High sensitive (hs-CRP) levels were analyzed as previously described [23]. Overall, for all the parameters the intra and inter-assay percentage coefficient and coefficient of variations were <3.0%, 1.9% and <5%, respectively.

### Ultrasound imaging

Presence of fat in liver was assessed with ultrasound using 3.5 MHz curvilinear probe (Siemens-G 60 S 2004, Germany). The definition of fatty liver was based on a comparative assessment of image brightness relative to the kidneys, in line with previously reported diagnostic criteria [24]. Severity of fatty liver was classified according to the brightness compared to kidneys, blurring of gall bladder wall, of hepatic veins and of portal vein. The radiologists performing the ultrasound were unaware of the clinical and laboratory results.

### Body composition

Percentage body fat (%BF), LBM and bone mineral density (BMD) were estimated by using whole body dual-energy X-ray absorptiometry (DEXA) scan (Lunar Prodigy Advanced Whole Body DEXA system, GE Medical Systems) as previously [5].

### Definitions

Overweight and obesity defined as BMI ≥23–24.9 kg/m^2^ and BMI ≥25 kg/m^2^, respectively according to criteria for Asian Indians [22]. Similarly, WC cut-offs of ≥90 cm for males and ≥80 cm for females were considered an indicator of abdominal obesity [25]. FBG≥100 mg/dl, serum TG ≥150 mg/dl (or on lipid lowering drugs), blood pressure >130/85 mmHg (or on antihypertensive therapy) and HDL-C; males ≤40 mg/dl, and females ≤50 mg/dl [26] were defined as abnormal. Insulin resistance was measured by two surrogate measures: fasting hyperinsulinemia and Homoeostasis Model Assessment of insulin resistance (HOMA-IR). The value of HOMA-IR was calculated as = fasting insulin (μU/ml) × fasting glucose (mmol/l)/22.5 [27]. Finally, hs-CRP level >1 mg/L was defined as high [22].

### Statistical Analysis

Data were entered in an Excel spreadsheet (Microsoft Corp, Washington, USA). The distribution of clinical, biochemical, anthropometry and body composition parameters was confirmed for approximate normality. We used mean and standard deviation to summarize the variables. The differences in biochemical anthropometry and body composition parameters in cases and controls were compared using the Student’s t-test. Difference between proportions was tested using Chi-square test. Bivariate logistic regression was performed to identify significant predictors of NAFLD. After adjusting for age, sex, TG, TC, HDL-C, LDL-C, WC, FBG, ALT, AST, ALK, fasting insulin, HOMA and % BF, multivariable logistic regression was carried out to identify the independent risk factor(s) and to estimate odds ratio (OR) and 95% confidence interval. For all above, a p value of <0.05 was considered as statistically significant.

We calculated the area under curve (AUC) for the receiver operating characteristic (ROC) for developing prediction equation for Asian Indians, henceforth termed as Indian Fatty Liver Index (IFLI). The performance of IFLI was compared with previously researched prediction equations in other populations as given below;

1. Fatty Liver Index (FLI) includes BMI, γ-GGT, TG, and WC [17]. FLI = (e0.953*loge (TG)+0.139 X BMI+0.718 X loge (GGT)+0.053X WC -15.745) / (1 + e 0.953 X loge (TG) + 0.139 X BMI + 0.718 X loge (GGT) + 0.053 X WC—15.745) X 100.
2. Lipid Accumulation Product (LAP) was calculated by expressing waist enlargement as the measured WC that exceeded a sex-specific minimum WC value and then multiplying it by fasting TG levels [18]. LAP for men = WC [cm]—65) × TG [mmol/L], and for women = WC [cm]—58) × TG [mmol/L].
3. NAFLD Liver Fat Score (LFS) includes AST/ALT ratio, T2DM, fasting AST level, fasting insulin level, and metabolic syndrome [19]. NAFLD liver fat score = -2.89+1.18 X metabolic syndrome (yes = 1/no = 0) + 0.45 X type 2 diabetes (yes = 2/no = 0) +0.15X fasting serum insulin (mU/L) + 0.04 X fasting AST (U/L)-0.94 XAST/ALT.
4. Liver Fat (%) includes metabolic syndrome, T2DM, fasting insulin, AST and AST/ALT ratio [19]. Liver Fat (%) = 10 (-0.805+0.282 X metabolic syndrome (yes = 1/no = 0) +0.078 X type 2 diabetes (yes = 2/no = 0)+ 0.525 X LOG (Fasting-insulin (mU/L)+ 0.521 X LOG (fasting AST (U/L)- 0.454 X LOG (AST/ALT))

Sensitivity, specificity, positive likelihood ratio (+LR), negative likelihood ratio (−LR), and corresponding 95% CIs were calculated for all equations for comparison. The non-invasive NAFLD measurement with the best performance (in terms of AUC for ROC) was then evaluated.

## Results

### Demographic, clinical, anthropometric, biochemical and body composition profiles

**Fig 1:**
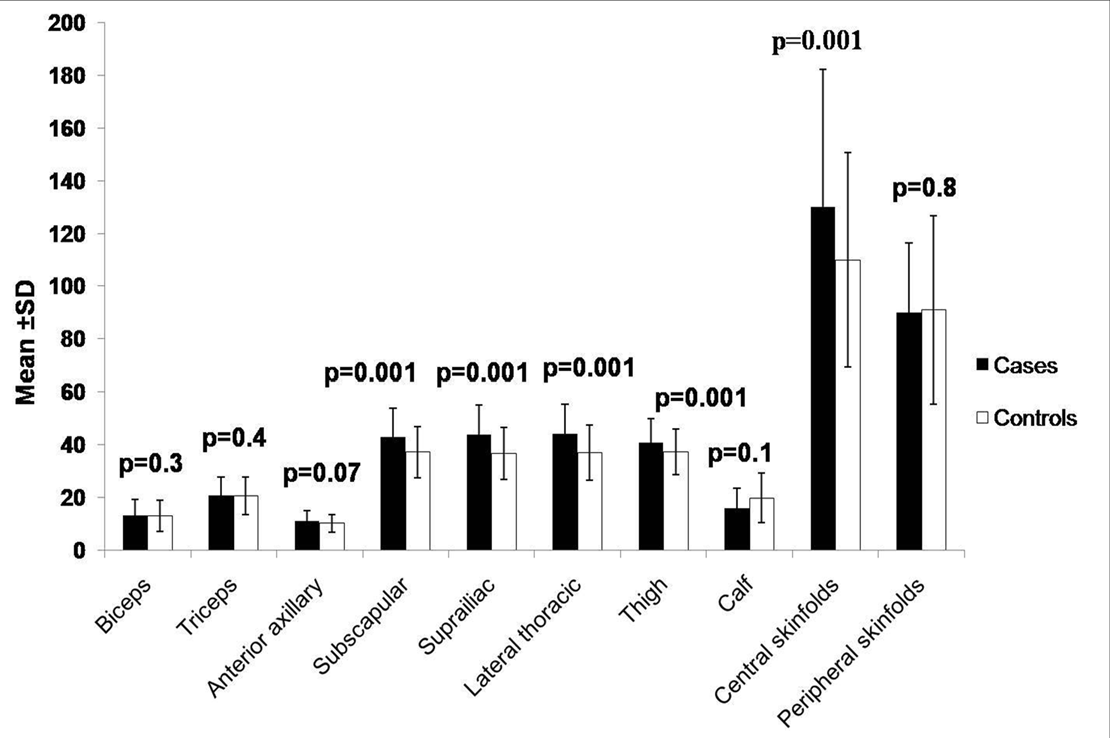
Comparison of skinfold thickness between cases (n = 162) and controls (n = 173). Central (sum of subscapular and suprailiac) and peripheral skinfold thicknesses (sum of biceps and triceps) were calculated.

Mean age of cases and controls) was similar. Buffalo hump, skin tags, xanthelasma, double chin, arcus, systolic blood pressure, diastolic blood pressure, BMI, WC, hip circumference (HC), waist hip ratio (WHR), mid thigh circumference (MTC), value of skinfolds; subscapular, suprailiac, lateral thoracic, thigh and central skin folds were significantly higher in cases as compared to controls (Table 1, Fig 1).

**Table 1:** Demographic, clinical and anthropometric profiles.

Variables	With NAFLD	Without NAFLD	p value
Age (yrs)	38.2±7.0	37.1±6.9	0.08
Sex* Male	129 (79.6)	109 (63.0)	0.001
Female	33 (20.3)	64 (36.9)	
Body mass index (kg/m^2^)	28.1±3.2	26.8±3.2	0.006
Waist circumference (cm)	94.5±9.4	89.8±9.1	0.001
Hip circumference (cm)	96.9±7.2	94.5±8.2	0.005
Waist-hip ratio	0.96±0.1	0.92±0.1	0.004
MTC (cm)	55.8±7.7	53.4±8.4	0.008
MAC (cm)	28.1± 3.3	27.4±5.7	0.1
Neck circumference (cm)	34.7 ± 4.2	33.9 ± 3.1	0.1
Acanthosis nigricans*	35 (21.60)	28 (16.1)	0.1
Buffalo hump*	47 (29.01)	20 (11.5)	0.001
Double chin*	82 (50.6)	51 (29.4)	0.001
Skin tags*	76 (47.20)	56 (32.3)	0.004
Xanthelasma*	15 (9.26)	4 (2.31)	0.005
Systolic blood pressure (mmHg)	125.0±11.8	119.3±11.0	0.001
Diastolic blood pressure (mmHg)	80.2±8.6	77.4±8.6	0.003

*All values are given as the number (%). P value <0.05 is statistically significant. Values are given as the mean ±standard deviation. MTC, mid thigh circumference; MAC, mid arm circumference

The levels of fasting blood glucose, serum TG, TC, LDL-C, VLDL, ALT, GGT, fasting insulin, HOMA-HR and hs-CRP were significantly higher in cases as compared to controls (Table 2). The intra and inter assay percentage coefficient variables were 2.30% and 1.92% for insulin and 2.03% and 1.56% for hs-CRP, respectively.

**Table 2:** Biochemical profile.

Variables	With NAFLD	without NAFLD	p value
Blood glucose (mg/dl)	89.7± 10.0	87.2± 10.8	0.04
Post-prandial blood glucose(mg/dl)┼	105.4± 10.9	104.7± 15.1	0.3
Total cholesterol (mg/dl)	189.1± 31.2	179.4± 26.8	0.002
Serum triglycerides (mg/dl)	172.0±78.0	148.0±65.3	0.002
HDL-C (mg/dl)	39.1± 6.2	39.3± 10.8	0.7
LDL-C (mg/dl)	110.5± 22.9	104.9± 24.1	0.03
VLDL (mg/dl)	33.4 ± 14.3	29.0 ± 14.0	0.01
ALT (IU/L)	38.7 ± 21.0	35.0 ± 13.7	0.05
AST (IU/L)	35.7 ± 19.2	33.6 ± 11.3	0.2
ALK (IU/L)	136.0 ± 57.5	135.2 ± 61.6	0.8
GGT (IU/L)	22.1±11.6	18.1± 6.8	0.0001
Insulin (μU/ml)*	9.7 (0.3–48.9)	6.7 (0.8–24.4)	0.0008
HOMA-IR*	2.5 (0.1–13.2)	1.6 (0.2–5.1)	0.009
Hs-CRP (μg/l) *	3.2 (0.03–14.3)	2.0 (0.25–13.5)	0.02

All values except that mentioned in line 2 are from fasting plasma levels. Values are given as the mean ±standard deviation.

* Wilcoxon rank-sum (Mann-Whitney) test, Median (minimum- maximum). P value <0.05 is statistically significant. LDL-C, low-density lipoprotein cholesterol; HDL-C, high-density lipoprotein cholesterol; VLDL, very-low density lipoprotein; ALT, alanine transaminase; AST, aspartate transaminase; GGT, γ glutamyl transpeptidase; HOMA-IR, homoeostasis modal assessment for insulin resistance; Hs-CRP, high sensitive C- reactive protein.

┼Blood taken 2 hours after first bite of standard breakfast.

**Fig 2:**
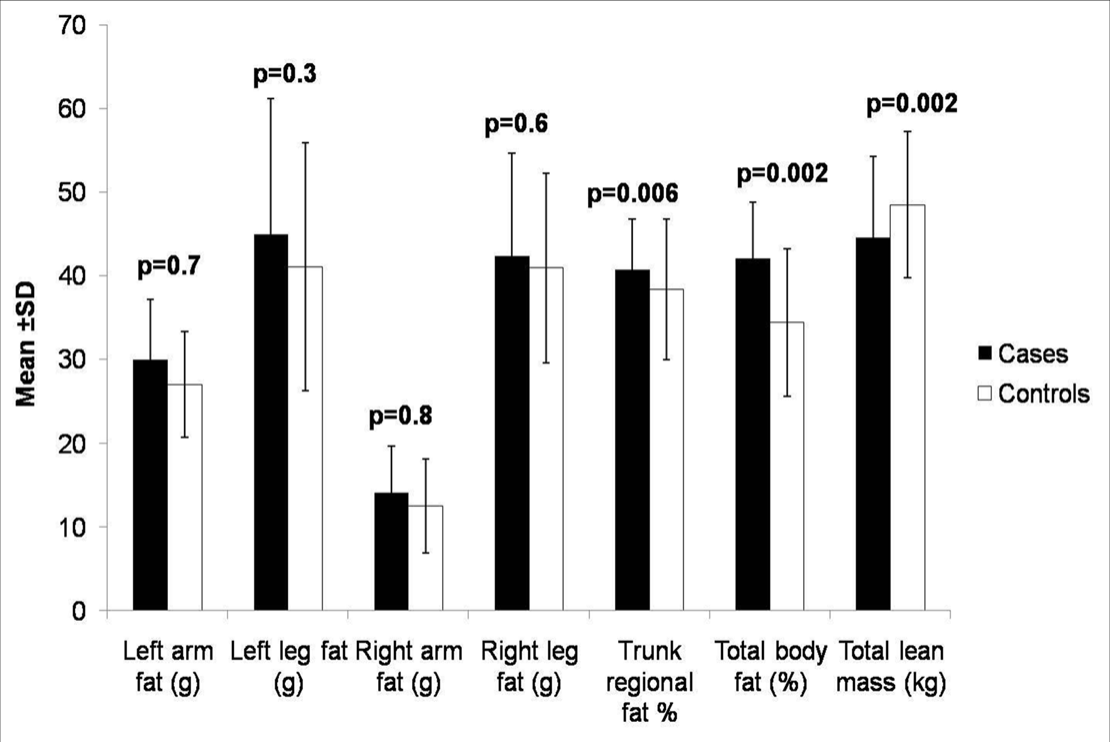
Body composition between cases (n = 162) and controls (n = 173) was estimated by using whole body dual-energy X-ray absorptiometry scan.

Body composition by Dual Energy X-ray Absorptiometry profiles were presented in Fig 2 and S1 Table. The values of right leg lean mass, right leg total mass, trunk fat percentage, trunk fat, total trunk mass, %BF and BF were significantly higher in cases as compared to controls, whereas % LBM and LBM were higher in controls.

### Univariate and step-wise logistic regression model

Using Univariate logistic regression model (Table 3), significant risk factors associated with the development of NAFLD were; double chin, buffalo hump, skin tag, xanthelasma, systolic blood pressure, WC, BMI, %BF, TG, fasting insulin, HOMA-IR and Hs-CRP. Using a step-wise logistic regression model as shown in Table 4, the significant factors associated with the development of NAFLD were double chin [2.2(1.21–7.0), p = 0.02], buffalo hump [2.94 (1.22–4.13), p = 0.01], systolic blood pressure [3.88 (0.97–10.4), p = 0.05], TG [2.33 (1.18–4.60), p = 0.01] and fasting Insulin [2.75 (1.38–5.50), p = 0.004].

**Table 3:** Univariate logistic regression analysis.

Variable	Unadjusted OR (95% CI)	p value
% Body fat	4.82 (2.4–9.71)	0.0001
Xanthelasma	4.31 (1.40–13.3)	0.004
Body mass index	3.33 (0.86–12.9)	0.05
Buffalo hump	3.13 (1.76–5.57)	0.0001
Fasting Insulin	3.03 (1.74–5.3)	0.001
hs-CRP	2.97 (1.08–8.2)	0.007
Serum triglycerides	2.68 (1.7–4.31)	0.001
Double chin	2.45 (1.56–3.84)	0.0001
Systolic blood pressure	2.0 (0.94–4.20)	0.05
Skin tag	1.87 (1.19–2.91)	0.005
Waist circumference	1.60 (1.02–2.50)	0.03
HOMA-IR	1.54 (0.91–2.64)	0.05

Hs-CRP, high sensitive C- reactive protein. HOMA-IR, homoeostasis modal assessment for insulin resistance

**Table 4:** Adjusted odds ratios (ORs) and 95% confidence intervals (CI) calculated using multivariate logistic regression analyses.

Variable	Adjusted OR (95% CI)	p value
Clinical		
Systolic blood pressure	3.88 (0.97–10.4)	0.05
Buffalo hump	2.94 (1.22–4.13)	0.01
Double chin	2.2 (1.21–7.0)	0.02
Biochemical		
Fasting Insulin	2.75 (1.38–5.50)	0.004
Serum triglycerides	2.33 (1.18–4.60)	0.01

Adjusted ORs were adjusted taking in consideration HOMA, hs CRP, diastolic blood pressure, weight, BMI, hip circumference, mid thigh circumference and total cholesterol.

### Prediction Models

**Fig 3:**
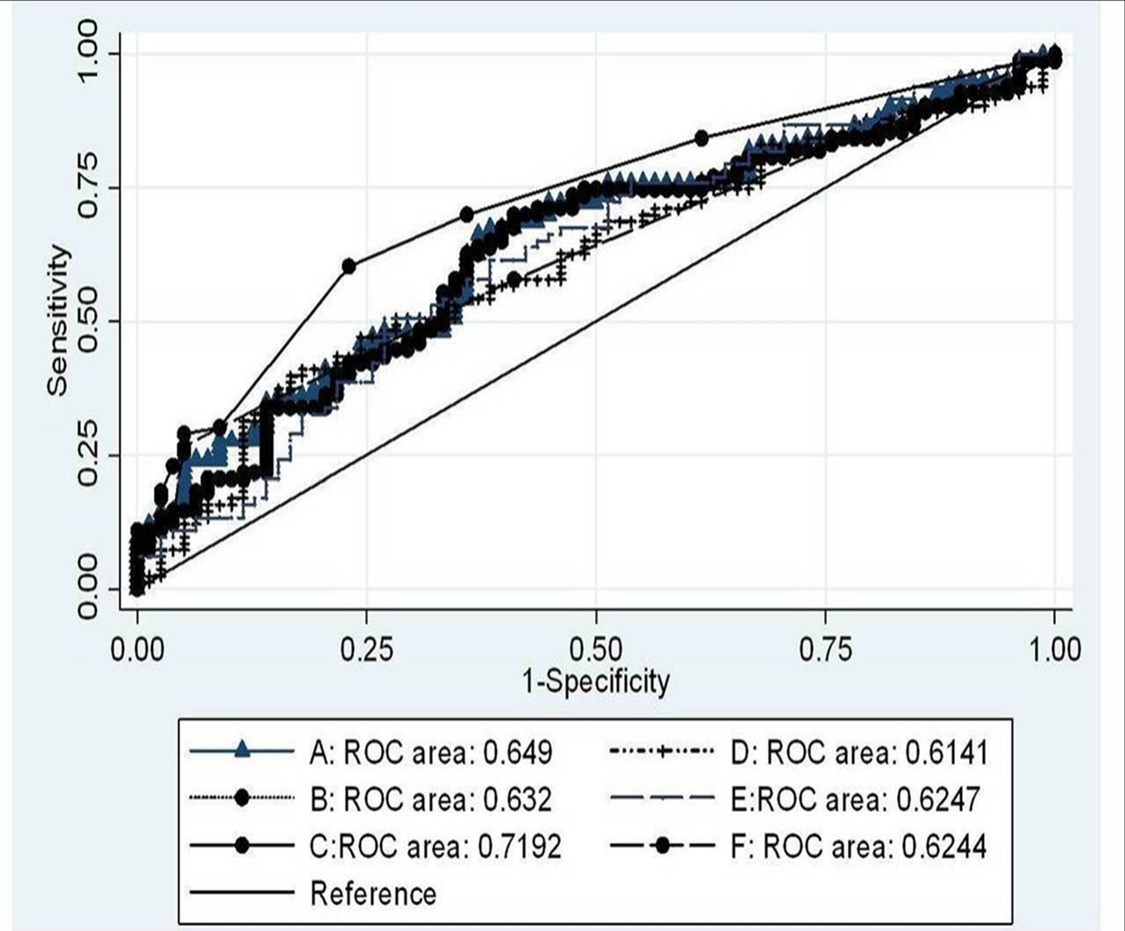
Area under curve (AUC) and 95% confidence interval (CI) for Indian Fatty Liver Index (IFLI), Fatty Liver Index (FLI), Lipid Accumulation Product (LAP), Liver Fat (LF) (%) and NAFLD Fat Score (NFS). A, IFLI-clinical; B, FLI; C, IFLI- clinical and biochemical; D, LAP; E, % LF and F, NFS.

1 Using Clinical variables (IFLI-C)
  Three clinical variables found to be significant in the logistic model were used to calculate the prediction score for NAFLD. The simplest equation for estimation of clinical parameters is: 1(double chin) +15.5(systolic blood pressure) +13.8(buffalo hump) (maximum score = 28.7, minimum score = 0). Systolic blood pressure: 1: (>120/80 mmHg); 0: otherwise. Using a ROC analysis (Fig 3) a score cut-off of ≥1.0 ensured the best balance between sensitivity and specificity. The sensitivity, specificity and ROC area under the curve (95% CI) were 64.81%, 61.85%, and 65.0 (59.0–70.67), respectively. The probability of subject having NAFLD was more if score was ≥1 with positive likelihood ratio = 1.67 and negative likelihood ratio = 0.58.

2 Using Clinical and Biochemical variable (IFLI-CB)
  Three clinical variables found to be significant in the logistic model were used to calculate the prediction score for NAFLD. The simplest equation for estimation of clinical and biochemical variables are: serum triglycerides +12(insulin) +16(systolic blood pressure) +18(buffalo hump) (maximum score = 47, minimum score = 0). Serum triglycerides: 1: (≥150 mg/dL); 0: otherwise; fasting Insulin: 1: (>2.7 μU/ml); 0: otherwise; systolic blood pressure: 1: (>120/80 mmHg); 0: otherwise. Using a ROC analysis (Fig 3) a score cut-off of ≥12 ensured the best balance between sensitivity and specificity. The sensitivity; specificity and ROC area under the curve (95% CI) were 64.29%, 66.81%, and 71.9 (65.09–78.1). The probability of subject having NAFLD was more if score was ≥28 (positive likelihood ratio = 2.09, negative likelihood ratio = 0.51).

Comparisons of IFLI-C and IFLI-CB with Previously Published Equations (Table 5)

**Table 5:** Comparison of Indian Fatty Liver Index, Fatty Liver Index, Lipid Accumulation Index, Liver Fat (%) and NAFLD Liver Fat Score prediction scores.

NAFLD Prediction score	AUC	95%Cl	Cut off	Sensitivity	Specificity	LR+	LR-
Indian Fatty Liver Index							
Clinical	65	59.1–70.67	≥1.0	64.2	61.85	1.67	0.58
CB	71.9	65.09–78.1	≥28	64.29	66.81	2.09	0.51
Fatty Liver Index^1^	63.2	47.8–64.4	≥99.25	57.28	57.65	1.35	0.74
Lipid Accumulation Product^2^	61.4	58.3–70.2	≥228.6	60.38	60.23	1.52	0.66
Liver Fat (%)^3^	62.4	55.1–69.8	≥858.13	60.71	60.18	1.52	0.66
NAFLD Liver Fat Score^3^	62.4	57.9–72.3	-0.714	62.5	62.83	1.71	0.67

All p values are <0.001. CB, Clinical and biochemical; AUC, area under curve; LR, likelihood ratio (+, positive; -; negative).

^1^n, 216 with and 280 without suspected liver disease; fatty liver was diagnosed by ultrasonography (17).

^2^n, 588; definition of fatty liver was based on liver ultrasonography (18).

^3^n, 359 non-diabetic, 111 type 2 diabetes; liver fat content was measured using proton magnetic resonance spectroscopy (19).

Importantly, ROC performances of IFLI-C (65.0%) and IFLI-CB (71.9%) were similar to NAFLD LFS but better than the FLI, LAP and Liver Fat (%).

## Discussion

This detailed phenotype analysis of patients with NAFLD shows adverse body composition features; high body fat, truncal fat, truncal subcutaneous fat, low lean mass, and ectopic fat deposition; excess fat deposition below chin (‘double chin’) and over the nape of the neck (‘buffalo hump’). Moreover, we present two prediction equations for NAFLD, one based on clinical parameters alone (IFLI-C), and other, a combination of clinical and biochemical parameters (IFLI-CB).

Overall, previous studies have shown that Asian Indians have more fat in various abdominal fat depots. In a comparative study of Asian Indians *vs*. white Caucasians in USA; for similar value of BMI, migrant Asian Indians had significantly greater total abdominal fat and intra-abdominal adipose tissue (IAAT) [28]. Some investigators have reported that truncal subcutaneous adipose tissue (SCAT; measured by subscapular and supra-iliac skinfolds and by magnetic resonance imaging) is thicker in South Asians than in White Caucasians [29, 30]. Recently, we have also shown that pancreatic volume (surrogate marker of pancreatic fat) and liver span (surrogate marker of liver fat) correlate strongly to diabetes in non-obese individuals [31]. Thicker truncal subcutaneous fat, as shown by skinfolds, and more truncal fat, signifies overall increased truncal and abdominal adiposity in persons with NAFLD in the current study. These increase fat depots, consisting mostly of metabolically dysfunctional adipocytes [32], generate increased amount of non-esterified fatty acids contributing significantly to fatty liver. It is important to note low lean mass in those with NAFLD, which may also of significance to glucose metabolism, and has been shown to have genetic basis in Asian Indians [33].

‘Buffalo hump’ and ‘double chin’, both signifying excess fat deposition at unusual sites, have been described by us as phenotypic markers closely correlating with metabolic syndrome in Asian Indians [15]. Incidentally, ‘buffalo hump’ is also present in HIV-associated lipodystrophy after prolonged use of protease inhibitors [34], and is associated with overall state of insulin resistance. Further, ‘double chin’ is commonly associated with diabetes in patients with partial lipodystrophies [34]. In the present study, both phenotypic markers were associated with NAFLD. Of significant note, both these signs could easily be detected on simple visual examination.

Interestingly, there are differences in the diagnostic prediction of different non-invasive scores, which may be accounted by sample of different populations studies and use of variegated phenotypic and biochemical measurements. Overall, assessment of phenotype done by us has been extensive, and includes conventional and novel signs (acanthosis nigricans, ‘buffalo hump’ ‘double chin’) based on previous studies done by others and by us. Many of these signs/markers have not been taken in account by other investigators while researching prediction equations. Further, some prediction equations include subjects with T2DM, which we have strictly excluded them, because many other variables in patients with T2DM may confound the prediction. These include changes the weight following diet and exercise; inclusion of drugs such as metformin *and* thiazolidinediones and vitamin E, all of which may have effect on liver fat.

It has been argued that other methods; magnetic resonance spectroscopy and liver biopsy are better tools for defining NAFLD, and could be considered as “gold standard”. Conversely, ultrasonography is by far the most common method of diagnosing NAFLD in clinical practice and has a fair sensitivity (87%) and specificity (94%) in detecting hepatic steatosis [35]. In a recent meta-analysis, forty-nine (4720 participants) studies regarding ultrasonography for diagnosis of fatty liver were included. Interestingly, the overall sensitivity, specificity, positive likelihood ratio, and negative likelihood ratio of ultrasound for the detection of moderate-severe fatty liver, compared to histology (gold standard), were 84.8% (95% confidence interval: 79.5–88.9), 93.6% (87.2–97.0), 13.3 (6.4–27.6), and 0.16 (0.12–0.22), respectively. Further, the area under the summary receiving operating characteristics curve was 0.93 (0.91–0.95). Overall, sensitivity and specificity of ultrasound was similar to that of other imaging techniques (i.e., computed tomography or magnetic resonance imaging) [36]. In summary, ultrasonography for liver fat is simple to perform, non-invasive, cost-effective and does not entail any radiation hazard, and could also be used in the epidemiological studies. Hence, although not “gold standard”, this method of investigation provides reasonable alternative to more expensive and difficult-to-perform diagnostic methods of NAFLD.

Two investigators have previously formulated prediction equations based on liver ultrasound estimated liver fat [17, 19]. In first study (prediction equation, FLI), 216 subjects with and 280 without NAFLD were studied [17], while in second study (prediction equation, LAP) 588 Italian adults were studied [18]. The performances of both the prediction equations on ROC curve analysis were inferior than IFLI-C and IFLI-CB presented by us. NAFLD Liver Fat score was developed using most robust estimation of liver fat using magnetic resonance spectroscopy in 470 Finnish subjects (non-diabetic and patients with T2DM) [19]. This score, however, performed better than IFLI on ROC curve analysis.

## Conclusion

Non-diabetic Asian Indians with NAFLD sampled by us, as compared to those without NAFLD, had adipose and insulin resistant phenotype. We also present prediction score for NAFLD; IFLI-C and IFLI-CB, which should be useful for clinicians and researchers.

## Supporting Information

S1 Table Body composition by Dual Energy X-ray Absorptiometry. (DOC)
