# Supplementary material for: Phenotype, Body Composition, and Prediction Equations (Indian Fatty Liver Index) for Non-Alcoholic Fatty Liver Disease in Non-Diabetic Asian Indians: A Case-Control Study
Source: PLoS One. 2015 Nov 24;10(11):e0142260. doi: 10.1371/journal.pone.0142260 (PMC4657982; doi:10.1371/journal.pone.0142260)
Supplement: S1 Table — (DOC) [file pone.0142260.s001.doc]

**Supplementary Table S1:** Body composition by Dual Energy X-ray Absorptiometry

Variables with NAFLD without NAFLD p value

Left arm fat (%) 30.2± 9.2 29.7±11.1 0.7

Left arm fat (g) 15.68±1.239 13.69± 6.98 0.07

Left arm lean mass (g) 31.13±8.12 30.20±782 0.2

Left arm total mass (kg) 4.8± 1.2 4.4±0.8 0.3

Left leg fat (%) 32.2± 8.4 33.1±10.3 0.3

Left leg fat (g) 44.92±2.9 41.12± 1.4 0.07

Left leg lean mass (g) 82.09±18.35 77.38±18.21 0.02

Left Leg total mass (kg) 13.0±2.1 12.3± 1.9 0.4

Right arm fat % 28.5±7.3 27.7±8.1 0.8

Right arm fat (g) 14.09± 5.4 13.05±6.1 0.3

Right arm lean mass (g) 31.92±7.6 30.20± 8.3 0.2

Right arm total mass (kg) 4.8±1.2 4.5± 1.9 0.4

Right leg fat % 31.2± 7.7 33.1± 10.3 0.6

Right leg fat (g) 42.34± 7.6 40.92±9.5 0.7

Right leg lean ass (g) 31.92±7.4 30.7±8.3 0.02

Right leg total mass (kg) 13±8.9 12.3±7.7 0.02

Trunk fat % 40.7±6.1 38.4±8.4 0.006

Trunk fat (g) 14.98±4.5 13.4± 42 0.001

Truncal lean mass (kg) 20.00±4.1 20.6±4.3 0.1

Total truncal mass (kg) 36.7±7.01 33.9±6.7 0.001

Total body fat (%) 35.1±6.7 32.3± 8.8 0.002

Total body fat (kg) 29.6±7.9 25.2±6.9 0.01

Total lean mass (%) 47.6±9.7 52.9±12.8 0.002

Total lean mass (kg) 49.4±8.2 54.2±7.7 0.04

Values are given as the mean ±standard deviation. P value <0.05 is statistically significant.
